# Innovative Chemical Pathways for CO_2_ Conversion

**DOI:** 10.3390/molecules31203694

**Published:** 2026-10-15

**Authors:** Guillermo Díaz-Sainz, Juqin Zeng

**Affiliations:** 1 Department of Chemical and Biomolecular Engineering, Universidad de Cantabria, 39005 Santander, Spain; 2 Department of Applied Science and Technology (DISAT), Politecnico di Torino, 10129 Turin, Italy; juqin.zeng@polito.it

The increasing concentration of atmospheric carbon dioxide (CO_2_) and the urgent need to mitigate anthropogenic greenhouse gas emissions have intensified scientific interest in developing sustainable carbon capture and utilization technologies. Beyond conventional emission reduction strategies, the conversion of CO_2_ into value-added chemicals, fuels, and other useful products offers opportunities to reduce dependence on fossil-based feedstocks and contribute to the transition towards a circular carbon economy [1,2]. Nevertheless, the thermodynamic stability of CO_2_, the complexity of competing reaction pathways, and the energy requirements associated with its activation represent considerable scientific and technological challenges. Furthermore, the environmental benefits of CO_2_ conversion depend strongly on the origin of the energy and reactants required, the efficiency of the conversion process, and the subsequent use of the resulting products [1,3]. Consequently, advances in catalytic materials, reaction engineering, and innovative conversion pathways must be accompanied by comprehensive assessments of their environmental and economic performance.

This Special Issue, titled “Innovative Chemical Pathways for CO_2_ Conversion”, brings together five original research articles and one review article addressing different approaches to carbon conversion and utilization. The contributions encompass the development of advanced catalysts for CO_2_ hydrogenation, plasma-assisted CO_2_ dissociation, molecular catalyst engineering for photo- and electrochemical CO_2_ reduction, biological CO_2_ fixation, and carbon monoxide generation from organic waste. Collectively, these contributions illustrate the diversity of approaches currently being explored to develop more sustainable carbon management strategies, highlighting the importance of combining advances in materials science and catalysis with improvements in reactor design and process optimization.

Among the different technologies for CO_2_ conversion, catalytic hydrogenation has attracted considerable attention because of its potential to produce methanol and methane, both of which have established applications in the chemical and energy sectors. Methanol is particularly interesting because of its versatility as a chemical intermediate, alternative fuel, and potential energy carrier. However, achieving high catalytic activity, product selectivity, and long-term operational stability remains challenging. Previous investigations have emphasized the importance of optimizing catalyst composition, metal–support interactions, and reaction conditions to improve methanol production via CO_2_ hydrogenation [4].

In this context, Figueroa-Quintero and co-workers [5] investigated the development of copper-based catalysts supported on modified NH_2_–MIL–125(Ti) metal–organic frameworks (MOFs) for CO_2_ hydrogenation to methanol. Through post-synthetic modification, titanium was partially replaced with zirconium or cerium to tune the interactions between the MOF support and the copper nanoparticles. The resulting materials were characterized and evaluated under different reaction conditions to establish relationships between catalyst composition and catalytic performance. Among the investigated materials, the catalyst incorporating titanium, zirconium, and copper exhibited the highest CO_2_ conversion and methanol selectivity, reaching values of approximately 5% and 60%, respectively, while largely suppressing methane formation. These findings highlight the potential of modifying MOF structures to control metal–support interactions and improve the selectivity of heterogeneous catalysts for CO_2_ hydrogenation.

The development of efficient catalysts for CO_2_ methanation represents another important research direction, particularly due to the potential of this process to be integrated with renewable hydrogen production and power-to-gas systems. Nickel-based catalysts are widely investigated for this application owing to their high catalytic activity and relatively low cost. Nevertheless, catalyst deactivation, metal sintering, and the influence of support properties remain important challenges. Understanding the relationships between catalyst structure, the nature and distribution of metallic active sites, and catalytic performance is therefore essential for developing more efficient and stable methanation catalysts [6].

Szymaszek-Wawryca and co-workers [7] investigated the preparation of high-loading nickel catalysts supported on metakaolin, a material derived from naturally occurring kaolin, for CO_2_ methanation. The catalysts, containing 30 wt.% nickel, were prepared by a melt infiltration method, and the influence of CeO_2_, MgO, and CaO as promoters on their physicochemical properties and catalytic performance was systematically evaluated. These promoters modified key catalyst properties, including NiO reducibility, metallic nickel dispersion, and textural characteristics. In particular, the addition of MgO increased the specific surface area and total pore volume compared with those of the unpromoted material. The promoted catalysts achieved approximately 80% CO_2_ conversion at 400 °C, approaching thermodynamic equilibrium, while maintaining methane selectivity above 97%. Furthermore, stable catalytic performance was observed during 24 h of continuous operation. This contribution highlights the potential of combining alternative mineral-derived supports with appropriate promoters to develop efficient and stable catalysts for CO_2_ methanation.

Beyond conventional thermocatalytic technologies, alternative activation methods are being explored to overcome some of the limitations associated with CO_2_ conversion. Non-thermal plasma represents a promising alternative, as energetic electrons can activate CO_2_ molecules while maintaining relatively low bulk gas temperatures. However, improving energy efficiency and gaining a deeper understanding of plasma–catalyst interactions remain essential challenges for the further development and potential industrial implementation of this technology [8].

Golubev and co-workers [9] investigated plasma-assisted CO_2_ dissociation into carbon monoxide using mesoporous cerium oxide catalysts in a dielectric barrier discharge reactor operating at near-room temperature. The catalysts were prepared using different synthesis methods and comprehensively characterized to establish relationships between their physicochemical properties and plasma-catalytic performance. The results demonstrated the importance of pore structure, surface basicity, and plasma–surface interactions in determining CO_2_ conversion efficiency. Among the investigated materials, the mesoporous CeO_2_ catalyst achieved a CO_2_ conversion of 32.3% at an input voltage of 5 kV, with carbon monoxide as the only detected reaction product and selectivity approaching 100%. Furthermore, no catalyst deactivation was observed during the stability tests. These findings highlight the potential of tailoring catalyst pore structure and surface properties to improve plasma-assisted CO_2_ dissociation, providing insights into the optimization of plasma-catalytic systems for CO_2_ conversion.

The rational design of molecular catalysts constitutes another promising strategy for promoting CO_2_ activation and controlling reaction pathways. Li and co-workers [10] presented a comprehensive review of recent advances in heteroatom-containing metalloporphyrin-based catalysts for CO_2_ reduction, with particular emphasis on N-confused and oxygen- or sulfur-substituted metalloporphyrins. Modifying the first coordination sphere of these molecular catalysts can alter the electronic properties of the catalytic metal center, thereby influencing the adsorption and stabilization of reaction intermediates and, consequently, catalytic activity and product selectivity. The review examined experimental and theoretical investigations involving photocatalytic and electrocatalytic CO_2_ reduction, highlighting the relationships between molecular structure, catalytic activity, and reaction mechanisms. Particular attention was given to elucidating the role of heteroatom incorporation and identifying opportunities for the rational design of novel molecular catalysts. These developments are especially relevant to advancing renewable electricity-driven chemical production, for which catalytic selectivity, energy efficiency, and long-term operational stability remain important considerations [11].

Biological CO_2_ fixation offers a complementary approach to carbon utilization by integrating carbon assimilation with the production of valuable biomass and bioproducts. In particular, microalgae have attracted considerable interest owing to their photosynthetic efficiency, potential for high biomass productivity, and ability to accumulate a wide range of valuable compounds with industrial applications. Previous investigations have emphasized the importance of optimizing photobioreactor design, light distribution, nutrient availability, and cultivation strategies to enhance CO_2_ fixation efficiency and overall process performance [12].

In this context, Tavares and co-workers [13] investigated the continuous cultivation of Haematococcus pluvialis using a two-stage photobioreactor system supplied with biogenic CO_2_ generated by a heterotrophic culture. The first stage operated as an autotrophic chemostat at different dilution rates, while the second stage incorporated high-irradiance columns to enhance biomass productivity and facilitate carotenoid accumulation. The integrated system achieved a maximum CO_2_ assimilation capacity of 0.747 g L^−1^ d^−1^ and a CO_2_ fixation efficiency of up to 94%. Furthermore, the two-stage configuration increased the daily CO_2_ assimilation rate by approximately 10% compared with the single-stage system. The authors also investigated the influence of cultivation conditions on biomass productivity, nutrient consumption, and pigment accumulation, demonstrating the importance of optimizing operational strategies to improve overall process performance. These results highlight the potential of integrating biological CO_2_ fixation with biomass valorization in sustainable biorefinery systems.

Complementing the approaches based on direct CO_2_ conversion and biological fixation, alternative strategies for obtaining carbon-containing chemical feedstocks from organic waste can contribute to broader carbon valorization efforts. Sobieraj [14] investigated carbon monoxide generation during food-waste composting, evaluating the effects of organic matter content and operating temperature in laboratory-scale bioreactors under oxygen-limited conditions. The experiments considered different food waste-to-gravelite ratios and temperatures ranging from 45 to 70 °C. The highest CO concentrations were obtained at 70 °C using a food waste-to-gravelite ratio of 1:2, corresponding to approximately 20% dry organic matter content. Under these conditions, average CO concentrations of 655 ppm and maximum concentrations of 2000 ppm were reported. The study also included preliminary estimates of potential industrial-scale CO production and its economic value. Although this approach does not involve the direct conversion of captured CO_2_, it provides a complementary perspective on carbon valorization by exploring the potential recovery of carbon monoxide generated during organic waste treatment. Further research is required to assess its practical feasibility, including CO recovery, purification, process integration, and environmental performance.

Taken together, the contributions presented in this Special Issue illustrate the diversity of scientific approaches being investigated for carbon conversion and utilization. They highlight the importance of understanding catalyst structure–performance relationships, tailoring the coordination environment catalytically active metal centers, integrating alternative activation methods, and optimizing biological carbon fixation processes. Furthermore, the reported studies emphasize the need to consider material properties, reaction mechanisms, and operating conditions collectively to improve the performance of carbon conversion and utilization technologies.

Despite these advances, several scientific and technological challenges remain before innovative CO_2_ conversion processes can achieve widespread industrial implementation. Further improvements in catalytic activity, product selectivity, energy efficiency, and long-term operational stability remain essential research priorities. In addition, future investigations should increasingly address reactor scale-up, process intensification, and the integration of these technologies with renewable energy sources and existing industrial infrastructure. Evaluating these technologies under realistic operating conditions, including the influence of impurities present in industrial CO_2_ streams, will be particularly important for assessing the practical applicability of emerging technologies.

The environmental and economic performance of CO_2_ conversion technologies must also be considered from the early stages of their development. The potential contribution of these processes to industrial decarbonization depends not only on conversion efficiency but also on the origin of the energy and reactants consumed, the environmental impacts associated with catalyst and equipment production, and the subsequent use and fate of the resulting products. Consequently, techno-economic assessments and life cycle assessments are essential tools for identifying technological limitations, establishing realistic performance targets, and quantifying potential environmental benefits [1,2,3,11].

In conclusion, this Special Issue highlights an overview of recent advances in innovative chemical and biological pathways for carbon conversion and utilization. The contributions demonstrate the importance of multidisciplinary research integrating chemistry, materials science, catalysis, biotechnology, chemical engineering, and sustainability assessment. We hope that these studies will stimulate further scientific discussion, encourage interdisciplinary collaboration, and support the development of increasingly efficient and sustainable technologies that contribute to industrial decarbonization and the transition towards a circular carbon economy.

We sincerely thank all the authors who contributed to this Special Issue, as well as the reviewers for their valuable comments and constructive evaluations. We also thank the editorial team of *Molecules* for their assistance and support throughout the editorial process.

## References

[1] Artz J., Müller T.E., Thenert K., Kleinekorte J., Meys R., Sternberg A., Bardow A., Leitner W. (2018). Sustainable Conversion of Carbon Dioxide: An Integrated Review of Catalysis and Life Cycle Assessment. Chem. Rev..

[2] Hepburn C., Adlen E., Beddington J., Carter E.A., Fuss S., Mac Dowell N., Minx J.C., Smith P., Williams C.K. (2019). The Technological and Economic Prospects for CO_2_ Utilization and Removal. Nature.

[3] Kätelhön A., Meys R., Deutz S., Suh S., Bardow A. (2019). Climate Change Mitigation Potential of Carbon Capture and Utilization in the Chemical Industry. Proc. Natl. Acad. Sci. USA.

[4] Navarro-Jaén S., Virginie M., Bonin J., Robert M., Wojcieszak R., Khodakov A.Y. (2021). Highlights and Challenges in the Selective Reduction of Carbon Dioxide to Methanol. Nat. Rev. Chem..

[5] Figueroa-Quintero L., Cordero-Lanzac T., Ramos-Fernandez E.V., Olsbye U., Narciso J. (2025). Tailoring Catalysts for CO_2_ Hydrogenation: Synthesis and Characterization of NH_2_–MIL–125 Frameworks. Molecules.

[6] Hu F., Ye R., Lu Z.H., Zhang R., Feng G. (2022). Structure–Activity Relationship of Ni-Based Catalysts toward CO_2_ Methanation: Recent Advances and Future Perspectives. Energy Fuels.

[7] Szymaszek-Wawryca A., Szymaszek M., Kosydar R., Duraczyńska D., Motak M. (2026). Tailoring the Structure and Surface Chemistry of High-Loading Ni-Metakaolin Catalysts Prepared by Melt Infiltration for CO_2_ Methanation. Molecules.

[8] Snoeckx R., Bogaerts A. (2017). Plasma Technology—A Novel Solution for CO_2_ Conversion?. Chem. Soc. Rev..

[9] Golubev O.V., Sadovnikov A.A., Maximov A.L. (2025). Plasma-Activated CO_2_ Dissociation to CO in Presence of CeO_2_ Mesoporous Catalysts. Molecules.

[10] Li Z., Wei Q., Ren Z., Xie J. (2025). Recent Progress in Heteroatom-Containing Metalloporphyrin-Based Catalysts for CO_2_ Reduction. Molecules.

[11] De Luna P., Hahn C., Higgins D., Jaffer S.A., Jaramillo T.F., Sargent E.H. (2019). What Would It Take for Renewably Powered Electrosynthesis to Displace Petrochemical Processes?. Science.

[12] Shekh A., Sharma A., Schenk P.M., Kumar G., Mudliar S. (2022). Microalgae Cultivation: Photobioreactors, CO_2_ Utilization, and Value-Added Products of Industrial Importance. J. Chem. Technol. Biotechnol..

[13] Tavares J., Paixão S.M., Silva T.P., Alves L. (2026). Enhanced CO_2_ Fixation Through Continuous Cultivation of Microalgae in a Two-Stage Photobioreactor System. Molecules.

[14] Sobieraj K. (2025). Optimization of the Organic Matter Content and Temperature in a Bioreactor to Enhance Carbon Monoxide Production During the Initial Phase of Food Waste Composting. Molecules.

